# Assessment of LES Subgrid-scale Models and Investigation of Hydrodynamic Behaviour for an Axisymmetrical Bluff Body Flow

**DOI:** 10.1007/s10494-016-9751-4

**Published:** 2016-07-30

**Authors:** Chin Yik Lee, Stewart Cant

**Affiliations:** Department of Engineering, University of Cambridge, Cambridge, UK

**Keywords:** Large eddy simulation, Axisymmetrical bluff-body flow, Subgrid scale models, Turbulent flow

## Abstract

This work is concerned with the investigation of fluid-mechanical behaviour and the performance of different subgrid-scale models for LES in the numerical prediction of a confined axisymmetrical bluff-body flow. Four subgrid-scale turbulence models comprising the Smagorinsky model, Dynamic Smagorinsky model, WALE model and subgrid turbulent kinetic energy model, are validated and compared directly against the experimental data. Two different mesh counts are used for the LES studies, one with a higher mesh resolution in the shear layer than the other. It is found that increasing the mesh resolution improves the time-averaged fluctuating velocity profiles, but has less effect on the time-averaged filtered velocity profiles. A comparison against experiment shows that the recirculation zone length is well predicted using LES. The accuracy of the four different subgrid scale models is then assessed by comparing the LES results using the dense mesh with the experiment. Comparisons with the time-averaged axial and radial velocity profiles demonstrate that LES displays good agreement with the experimental data, with the essential flow features captured both qualitative and quantitatively. The subgrid velocity also matches well with the experimental results, but a slight underprediction of the inner shear layer is observed for all subgrid models. In general, it is found that the Smagorinsky and WALE models are more dissipative than the Dynamic Smagorinsky model and subgrid TKE model. Comparison of the spectra against the experiment shows that LES can capture dominant features of the turbulent flow with reasonable accuracy, and weak spectral peaks related to the Kevin-Helmholtz instability and helical vortex shedding are present.

## Introduction

Bluff-body flows are of particular importance in many applications, including aerodynamic drag reduction in vehicles [1], transitional flow separation in airfoils [2] or for flame stabilization in combustion systems [3, 4]. At low Reynolds number, analysis of axisymmetrical bluff-body flows such as spheres or disks using hydrodynamic stability theory [5, 6] and Direct Numerical Simulation (DNS) [7] has revealed detailed insight into the flow structure and aided in the understanding of the dynamics of bluff-body flows. The nature of this kind of flow is such that an oscillatory coherent flow structure tends to develop from a relatively steady boundary condition, even in a symmetric geometry. Also present is the well-known phenomenon of helical vortex shedding behind bluff-bodies occurring at distinct Strouhal number, which has been extensively investigated both experimentally [8, 9] and numerically [10]. The challenge in the prediction or such flows arise at higher Reynolds number when the flow starts transitioning to turbulence through multiple bifurcations and eventually various instabilities set in [11, 12]. Despite the complexity associated with such flows, the understanding and prediction of bluff-body flow is of paramount importance.

In this work, the main motivation to study bluff-body flow lies in its ability to stabilize flames. The flow features behind the asymmetric bluff body including the shear layer, recirculation zone and sudden expansion facilitate flame stabilization [4]. The recirculation zone carries burnt gases at high temperature that act as a source of continuous heating of unburned reactants and creates a low velocity region that enables a stable flame to be anchored on the bluff-body under high Reynolds number conditions. Such flows are commonly observed and studied in model and realistic aeroengine combustion chambers. In order to stabilize the flame successfully, a steady flow is required [13]. Yet, experiment and previous work suggest that coherent structures can develop even under such conditions. Further, the evolution and decay of larger structures can lead to smaller vortical structures of different length-scales which have to be appropriately represented to capture the underlying dynamics of the flow. The presence of these structures would have an implication on the stabilization mechanism and flame structure. Gaining a further insight of these flow structures would therefore be useful. The use of DNS to study such flows remains expensive, and hence the computation may be limited to low Reynolds numbers and short flow times. In this work, the isothermal flow behaviour for the asymmetric bluff body is investigated using Large Eddy Simulation (LES). In LES, a large range of scales contained in the flow is explicitly resolved, and the smaller scale features are modelled [14]. Under conditions where large-scale vortices are present, LES holds advantages over traditional approaches such as Reynolds-averaged Navier-Stokes (URANS). LES is also capable of representing highly unsteady and transient phenomena.

The configuration investigated in this study is a confined axisymmetric bluff body flow in an experimental rig developed in Cambridge University to study the response of both turbulent isothermal and reacting flow under acoustic forcing [15]. The key objective is to verify that LES is capable of representing the axisymmetric non-reacting bluff-body flow features with reasonable accuracy. This is important as the ultimate goal of the simulation is to predict self-excited combustion oscillation [16] which is commonly observed in combustion systems when the heat release and pressure fluctuations are in-phase. It is well established that intrinsic instabilities associated with underlying hydrodynamics in the system [17, 18] act as a key mechanism for combustion oscillation. Changes in the flow modify the flame structure and can also act as a coupling mechanism for self-excited oscillation [19]. Both experimental [15, 20] and numerical studies [21–23] on this geometry are available, but the primary focus of most of this previous work is to characterize the flame response to inlet flow perturbations and to understand the flame dynamics. A non-reacting flow study was previously conducted by Ayache et al. [24] using LES to examine the flow structure and the flow response to inlet forcing. Isothermal studies serve as useful first steps in understanding the fluid mechanics in burners and demonstrating that the flow field is well represented before the reacting model is applied. This is of particular importance in order to capture the correct recirculation zone length and identify the presence of any flow instabilities in the system.

The aim of this work is to assess and compare the accuracy of different subgrid-scale models in the prediction of an turbulent axisymmetric bluff-body flow, and establish whether the fundamental flow features in this system can be adequately captured. A commercially available CFD toolkit OpenFOAM (Open Field Operation and Manipulation) [25] is used. The paper is organized as follows. LES results obtained using the Smagorinsky model are first compared against experimental and numerical results from previous studies. Two different mesh configurations are examined to seek an optimum mesh for the current setup and to evaluate the effects of shear development close to the bluff-body. Detailed comparisons are then carried out using the experimental data, including smoke visualization and the axial and radial velocity profiles taken at different locations, with particular focus on the second moment quantities. Next, some commonly-used LES subgrid models, including the Smagorinsky model, Dynamic Smagorinsky model, WALE model and subgrid turbulent kinetic energy (TKE) model are compared and assessed. The spectra and probability density function (pdf) of the local velocity are chosen as the criteria for checking the accuracy and resolution of the simulation. The overall flow structure is examined and in particular the effect of the subgrid-scale model on the prediction of flow patterns is addressed. The dominant features of the flow are illustrated and used to establish consistency in the results for the different subgrid models employed. The flow instabilities in the system are also briefly discussed.

## Large Eddy Simulation

In LES, the larger scales are resolved directly and the smaller scales are modelled [26]. A low-pass filter is applied to separate the resolved and subgrid scale. To account for the acoustic effects on the flow, a compressible formulation is used. The filtered continuity, momentum and energy equations are:

1 $$ \frac{\partial{\bar{\rho}}}{\partial{t}}+\frac{\partial({\bar{\rho}}\tilde{u}_{i})}{\partial{x_{i}}}=0 $$

2 $$ \frac{\partial{(\bar{\rho}}{\tilde{u}_{j}})}{\partial{t}}+\frac{\partial{(\bar{\rho}}\tilde{u}_{i}\tilde{u}_{j})}{\partial{x_{i}}}=-\frac{\partial{\bar{p}}}{\partial{x_{j}}}+\frac{\partial{\overline{\tau}_{ij}}}{\partial{x_{i}}}-\frac{\partial}{\partial{x_{i}}}[\overline{\rho}(\widetilde{u_{i}u_{j}}-\tilde{u}_{i}\tilde{u}_{j})] $$

3 $$ \frac{\partial{(\bar{\rho}}{\tilde{e}})}{\partial{t}}+\frac{\partial{(\bar{\rho}}\tilde{u}_{i}\tilde{e})}{\partial{x_{i}}}= -{\frac{\partial}{\partial{x_{i}}}(\overline{p}\tilde{u}_{i})} -\frac{\partial{\overline{q_{i}}}}{\partial{x_{i}}} +{\frac{\partial}{\partial{x_{j}}}(\tilde{u}_{i}\overline{\tau}_{ij})} -\frac{\partial}{\partial{x_{i}}}[\overline{\rho}(\widetilde{u_{i}e}-\tilde{u}_{i}\tilde{e})] $$

where ($\tilde {}$) denotes Favre-filtering and ($\bar { }$) denotes the filtering operation. The Favre-filtered velocity in the *i* th direction is $\tilde {u}_{i}$, $\tilde {e}$ is the internal energy, $\bar {\rho }$ is the resolved density and $\bar {\tau }_{ij}$ is the resolved stress tensor. In the equations above, the unclosed terms are the subgrid scale stresses $(\widetilde {u_{i}u_{j}}-\tilde {u}_{i}\tilde {u}_{j})$ and scalar fluxes for the internal energy $(\widetilde {u_{i}e}-\tilde {u}_{i}\tilde {e})$. The unresolved scalar fluxes are treated using a standard gradient assumption [27]. Modelling of the subgrid stress terms using different subgrid-scale models will be described in next section.

## Modelling Subgrid Stresses

### Smagorinsky-Lilly model

For the Smagorinsky model [28], the anistropic subgrid scale stress tensor is evaluated as

4 $$ \tau^{s}_{ij}-\frac{1}{3}\delta_{ij}\tau^{s}_{kk}=-2\mu_{t}\left(\tilde{S}_{ij}-\frac{\delta_{ij}}{3}\tilde{S}_{kk}\right)  $$

The isotropic part of the viscous and subgrid stresses $\tau _{kk}^{s}$ is absorbed into the filtered pressure. The expression *μ*_*t*_ is the subgrid eddy viscosity, $\tilde {S}_{ij}$ is the Favre filtered rate of strain tensor and $\tilde {S}_{kk}$ is the Favre filtered trace of the strain tensor. The subgrid-scale viscosity *μ*_*t*_ is expressed as

5 $$ \mu_{t}=\bar{\rho}(C_{s}\Delta)^{2} |\tilde{S}_{ij}|  $$

The model constant *C*_*s*_ takes a value of 0.15. This value was found adequate for the current flow configuration, and was selected through a preliminary study where different values of *C*_*s*_ were tested. When using a value of 0.18, excessive damping of the resolved turbulence and high subgrid scale turbulent kinetic energy are found, affecting the recirculation zone length predicted from LES as compared to experiment. Conversely, the value of 0.1 was found to be too low, resulting in an underprediction of turbulent kinetic energy in the shear layers. The filter width Δ is taken as the cube root of the local grid cell volume. The Favre filtered rate of strain tensor $\tilde {S}_{ij}$ is defined as

6 $$ \tilde{S}_{ij}=\frac{1}{2}\left(\frac{\partial \tilde{u}_{j}}{\partial x_{i}}+\frac{\partial \tilde{u}_{i}}{\partial x_{j}}\right)  $$

The term $|\tilde {S}_{ij}|$ represents the Frobenius form of the Favre filtered rate of strain tensor *S*_*ij*_ and takes the form

7 $$ |\tilde{S}_{ij}|=\sqrt{2 \tilde{S}_{i} \tilde{S}_{j}}  $$

### Dynamic Smagorinsky model

The Dynamic Smagorinsky model [29] uses the resolved scales to evaluate the model coefficient *C*_*s*_ during the simulation, thus avoiding the need to tune the coefficient a priori. The dynamic procedure in this model is based on the Germano identity, where the resolved turbulent stress tensor *L*_*ij*_ takes the form:

8 $$ L_{ij}=T_{ij}^{s}-\widehat{\tau}_{ij}^{s}  $$

where ($\hat {}$) denotes a test filter. The subgrid stress tensor for the test filter scale $T_{ij}^{s}$ is defined as

9 $$ T_{ij}^{s}=\widehat{\widetilde{u_{i}u_{j}}}-{\hat{\tilde{u}}}_{i}{\hat{\tilde{u}}}_{j}  $$

The test filtered subgrid stress tensor for the filter scale $\tau _{ij}^{s}$ is defined as

10 $$ \tau_{ij}^{s}=\widehat{\widetilde{u_{i}u_{j}}}-{\widehat{\tilde{u}_{i}\tilde{u}_{j}}}  $$

The test filter $\hat {\Delta }$ is larger than the grid filter Δ. Equation 8 should hold for all time and space but can become ill-conditioned as this formulation leads to an overdetermined system. Here, the least-squares error method [30] is used to treat this problem. The equation for *C*_*s*_ takes the form:

11 $$ {C_{s}^{2}}=\frac{\left<L_{ij}M_{ij}\right>}{\left<M_{ij}M_{ij}\right>}  $$

where

12 $$ M_{ij}=2\Delta^{2}\left(\widetilde{|\hat{S}_{ij}|\hat{S}_{ij}}-\alpha^{2}|\hat{\tilde{S}}_{ij}|\hat{\tilde{S}}_{ij}\right)  $$

where *α* is the ratio of the test filter to the grid filter $\hat {\Delta }/\Delta $.

### WALE model

The Wall-Adapting Local Eddy-viscosity (WALE) model [31] focuses on capturing near wall behaviour for confined flows for complex geometry. The subgrid eddy viscosity in the WALE model is modelled as

13 $$ \mu_{t}=\overline{\rho}(C_{w}\Delta)^{2}\frac{\left({S_{ij}^{d}}{S_{ij}^{d}}\right)^{3/2}}{(\tilde{S}_{ij}\tilde{S}_{ij})^{5/2}+\left(S_{ij}^{d}S_{ij}^{d}\right)^{5/4}}  $$

where the constant *C*_*w*_ is taken as 0.5, $\tilde {S}$ is the filtered rate of strain tensor and $S_{ij}^{d}$ is the traceless symmetric part of the square of the velocity gradient tensor *g*_*ij*_, defined as:

14 $$ S_{ij}^{d}=\frac{1}{2}\left(\tilde{g}_{ij}^{2}+\tilde{g}_{ji}^{2}\right)-\frac{1}{3}\delta_{ij}\tilde{g}_{kk}^{2}  $$

where *g*_*ij*_ denotes *∂* *u*_*i*_/*∂* *x*_*j*_.

### One-equation turbulent kinetic energy model

The subgrid-scale kinetic energy [32, 33] is defined as:

15 $$ k_{\text{sgs}} = \frac{1}{2}\left(\widetilde{{u_{k}^{2}}} - \tilde{u}_{k}^{2} \right) $$

In the one-equation model, the transport equation for subgrid-scale kinetic energy is solved.

16 $$ \frac{\partial \tilde k_{\text{sgs}}}{\partial t} + \frac{\partial \tilde u_{j} k_{sgs}} {\partial x_{j}} = \frac{\partial}{\partial x_{j}} \left(\frac{\mu_{t}}{\sigma_{k}} \frac{\partial k_{\text{sgs}}}{\partial x_{j}} \right) - \tau_{ij} \frac{\partial \tilde u_{i}}{\partial x_{j}} - C_{\varepsilon} \frac{k_{\text{sgs}}^{3/2}}{\Delta} $$

where the constants *C*_*ε*_ and *σ*_*k*_ are taken as 0.916 and 1.0, respectively. The subgrid-scale stress can be expressed as

17 $$ \tau_{ij} =-2 \mu_{t}\tilde {S}_{ij} + \frac{2}{3} k_{\text{sgs}} \delta_{ij} $$

The subgrid-scale eddy viscosity *μ*_*t*_ is computed using *k*_sgs_ as

18 $$ \mu_{t} = C_{k} k_{\text{sgs}}^{1/2} \Delta $$

where *C*_*k*_ is taken as 0.09.

## Numerical Configuration

**Fig. 1:**
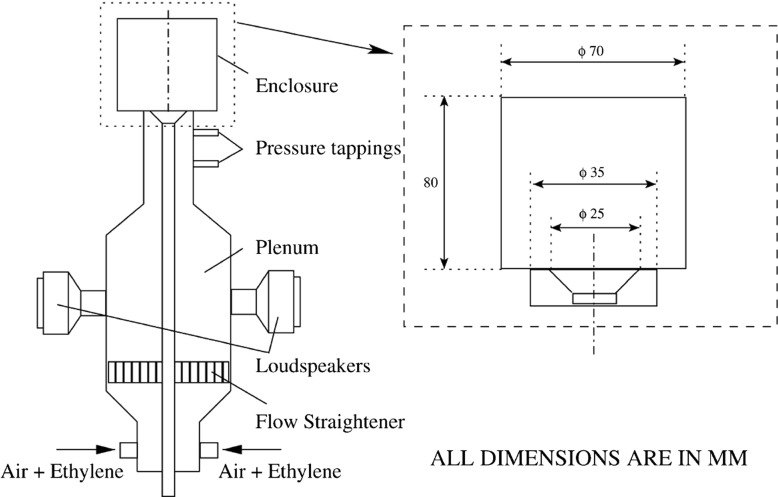
Experimental setup for axisymmetrical bluff-body enclosed flow, Balachandran et al. [20]

**Fig. 2:**
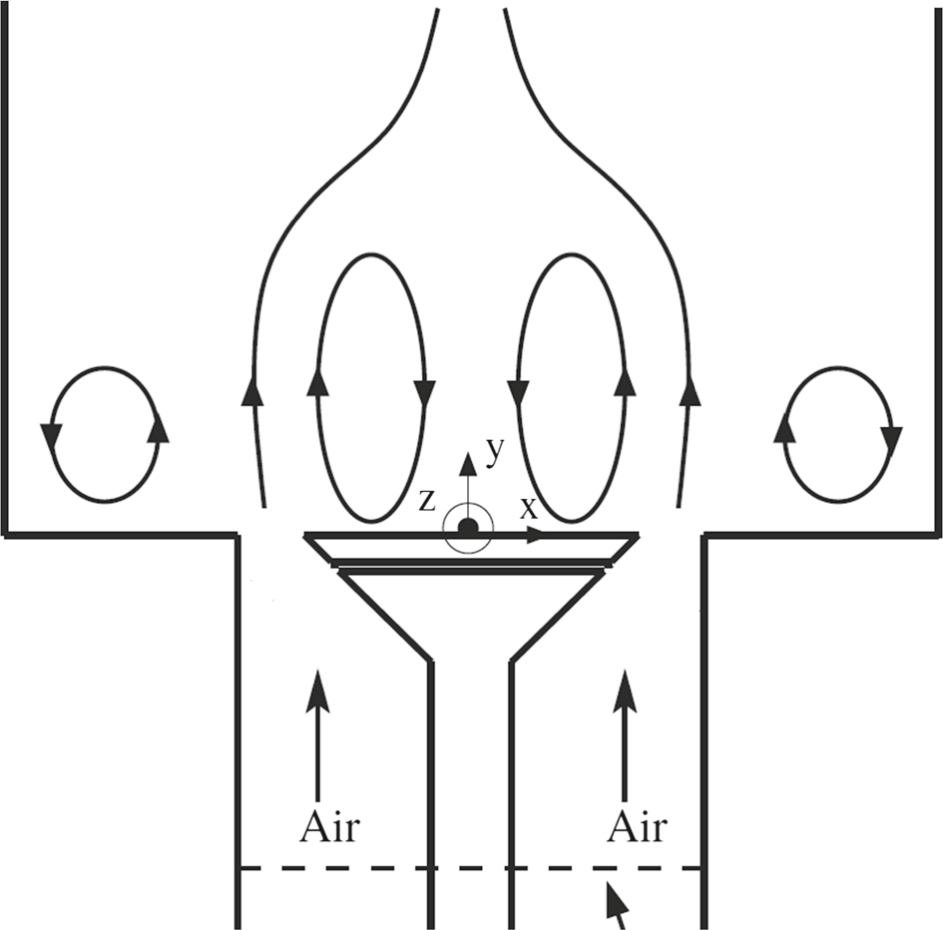
Schematic of the enclosure showing the air inlet, the central recirculation zone and the side recirculation zone

The experimental setup is shown in Fig. 1. This experiment [20] was designed to study oscillating flames and investigate inert flow behaviour at ambient conditions. The rig consists of a long circular duct of internal diameter 35 mm with a conical bluff-body of diameter *D*_*b*_ equal to 25 mm. The angle of the bluff body is 45 degrees (half angle), giving a blockage ratio of 50 *%*. The enclosure is a 80 mm long quartz cylinder with internal diameter 70 mm to provide optical access for velocity measurement and to avoid air entrainment. The region upstream of the rig consists of a plenum chamber and loudspeakers to facilitate acoustic forcing. These will not be considered in the current study as the flow investigated is not subjected to external acoustic forcing. The time-averaged bulk inlet velocity *U*_*b*_ of the flow is 10 m/s. This gives a Reynolds number Re = *U*_*b*_ *d*/*ν* of 16000, where *d* is the bluff-body diameter and *ν* is the kinematic viscosity. The general flow structure in the experiment is shown in Fig. 2. Different features of the flow can be distinguished, including two (left and right) inlet flows that leads to opposing annular jets and their corresponding shear layers, the central and outer recirculation zones and a forward stagnation point at the bluff body wake.

**Fig. 3:**
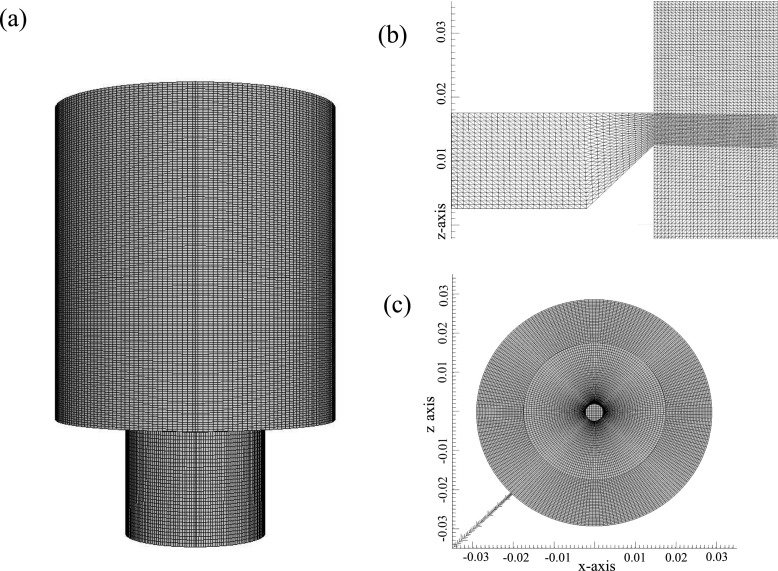
Mesh layout of the computational domain for the bluff-body flow. General features (**a**), bluff body region (**b**) and bottom view (**c**)

In the numerical simulation, the full enclosure extending up to 30 mm upstream of the bluff body is simulated, allowing for the flow development (Fig. 2). The mesh layout of the computational domain is shown in Fig. 3. A multi-block mesh is constructed using two different mesh counts of 3.0 million and 4.0 million cells, respectively. The key difference between the mesh configurations is in the mesh refinement along the separated shear layer of the bluff body. This mesh resolution in the shear layer is related to that along the wall upstream behind the bluff-body and is therefore important to capture the development of the shear layer.

For the LES, an inlet velocity of 5.15 m/s is prescribed such that the mass balance is satisfied between the inlet of the computational domain and the location where the flow enters the enclosure at the annular opening to achieve a mean velocity of 10 m/s. The temperature at the inlet is fixed at 288 K. The inflow turbulence is prescribed using the Synthetic Eddy Method (SEM) [34] with an amplitude of 5 *%* of the mean velocity. The solid walls are treated as adiabatic and impermeable. A wave-transmissive outlet boundary condition [35] is employed at the outlet. The damping parameter [35] is set to be small so that the boundary can be regarded as partially reflecting.

The models are implemented in the open source CFD toolkit OpenFOAM [25]. The equations are discretized in space using a central differencing scheme which is second-order accurate. A second-order backward time marching scheme is employed to account for transient effects. The system of equations is solved using the Pressure Implicit with Splitting of Operators (PISO) pressure correction algorithm [36]. The algorithm splits the solution procedure into an implicit predictor step followed by two corrector steps. In the predictor step, all quantities except the pressure are updated by solving the momentum and energy equations implicitly, whilst in the corrector steps, the pressure Poisson equation is solved implicitly and other quantities are updated explicitly.

For these studies, an adaptive timestep in the range of 1−2 × 10^−7^ s has been used. These values ensure a Courant–Friedrichs–Lewy (CFL) number less than 0.1 everywhere in the domain. The LES simulations are run for at least 30 characteristic time periods *τ*_*b*_ = *D*_*b*_/ *U*_*b*_ before the statistics are collected. The collected data are gathered over 40 *τ*_*b*_ to obtain the results for the mean and second moment quantities.

## Results and Discussion

### Comparison of fundamental flow features

**Fig. 4:**
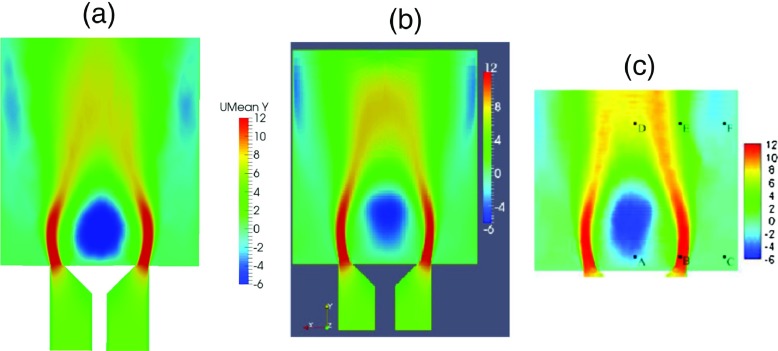
Time-averaged axial velocity contours for (**a**) LES and results from previous investigations by (**b**) Han and Morgans. [23] and (**c**) Ayache et al. [24]

In this section, the CFD results from LES (Smagorinsky model) are first compared against previous work. Figure 4 shows the time-averaged axial velocity profiles for LES (a) and results from previous studies by Han and Morgans [23] using the WALE model (b) and Ayache et al. [24] using the Dynamic Smagorinsky model (c). It can be seen that the time-averaged axial velocity from the current study exhibits good qualitative and reasonable quantitative agreement with earlier LES investigations.

**Fig. 5:**
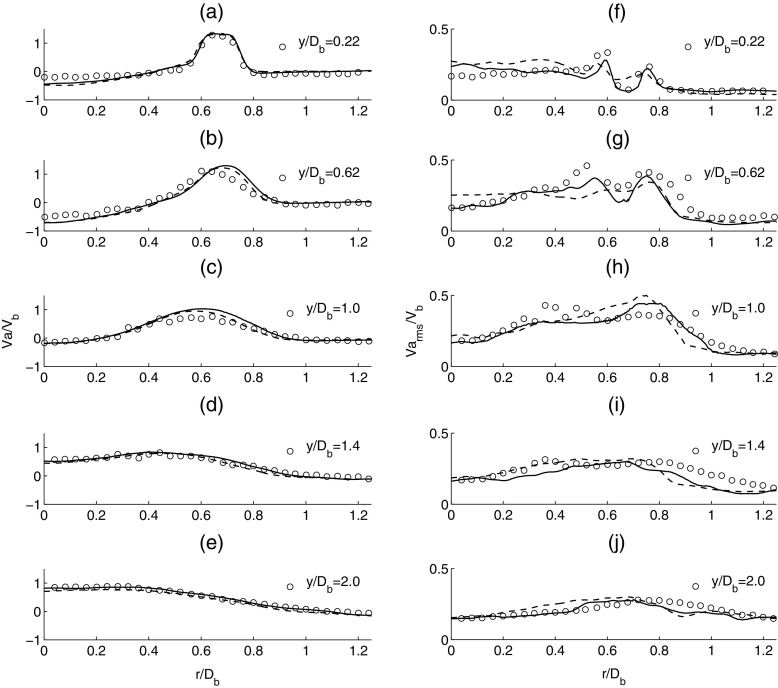
Time-averaged mean axial velocity (*left column*: **a-e**) and time-averaged rms axial velocity (*right column*: **f-j**). Experiment (*circle*), solid line (*dense mesh*) and dashed line (*coarse mesh*)

In order to assess the accuracy of the simulation results, a direct comparison with the experiment is performed. The comparison is made against the mean and root mean square (rms) of both the axial and radial velocity profiles from the experiment. The effect of mesh resolution is first investigated by comparing the results for the coarse and dense grids for LES using the Smagorinsky model. Figure 5 presents the comparison for the the mean (a-e) and rms (f-j) axial velocity profiles. The mean and rms velocities are normalized against the inlet velocity *V*_*b*_ and the radial distance is normalized against the bluff body diameter *D*_*b*_. The velocity profiles are obtained at five different locations in the experiment corresponding to *y*/*D*_*b*_ of 0.22, 0.62, 1.0, 1.4 and 2.0 from the annulus, where *y* is the axial distance and *D*_*b*_ is the diameter of the bluff-body. For the LES, good agreement with the experiment is obtained for the mean axial velocity profiles for both coarse and dense grids. The negative axial velocity close to the centreline in Fig. 5 indicates that the stagnation zone extends further downstream from *y*/*D*_*b*_ = 1.0. It can also be observed that the jet strength decreases from the inlet to the outlet and an acceleration close to the centreline occurs in the downstream wake due to sudden expansion. Comparison of the second moment quantities for the axial velocity (f-j) clearly demonstrates that the LES with the dense mesh provides a better representation of the time-averaged axial velocity fluctuation profiles from the experiment when compared against the coarse mesh solution. The velocity fluctuations attain their highest value at the exit of the bluff-body and they are equal to approximately 15 *%* of *V*_*b*_ inside the recirculation zone.

**Fig. 6:**
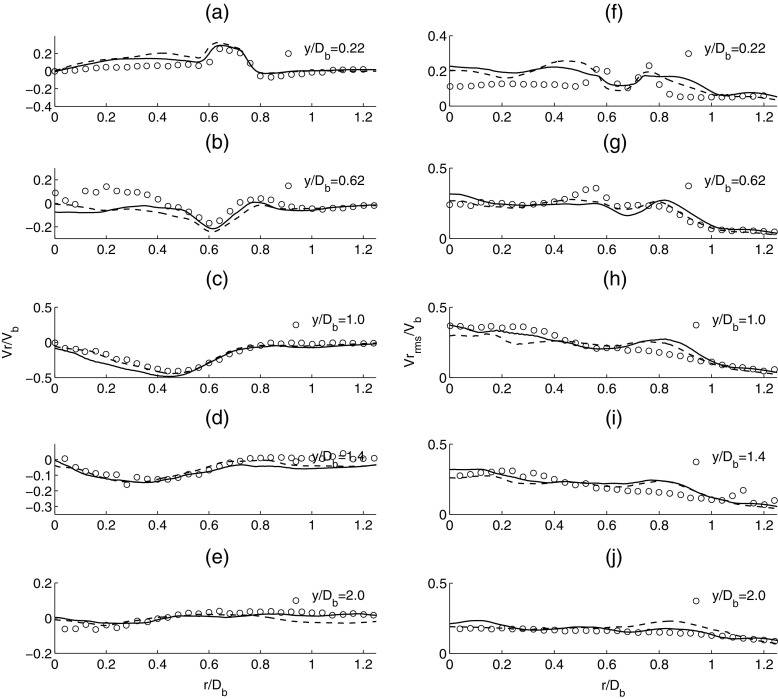
Time-averaged mean radial velocity (*left column*: **a-e**) and time-averaged rms radial velocity (*right column*: **f-j**). Experiment (*circle*), solid line (*dense mesh*) and dashed line (*coarse mesh*)

The comparisons for the mean and rms radial velocity are shown in Fig. 6. For the mean radial velocity (a-e), the LES results are almost independent of the mesh used and match well with the experiment. This suggests that the development of the recirculation zone and vortex stretching effects in the azimuthal direction are adequately represented in LES. For the second moment quantities (f-j), LES provides good agreement with the experimental data. The LES predictions of the flow profile close to the centreline exhibit some discrepancies close to the shear layer but is found to improve in the downstream region. Based on the comparisons from Figs. 5 and 6, it is found that LES provides a good prediction of the mean velocity profiles. A clear improvement in the profiles of the rms velocities compared with the experiment can be seen when the mesh resolution is increased.

**Fig. 7:**
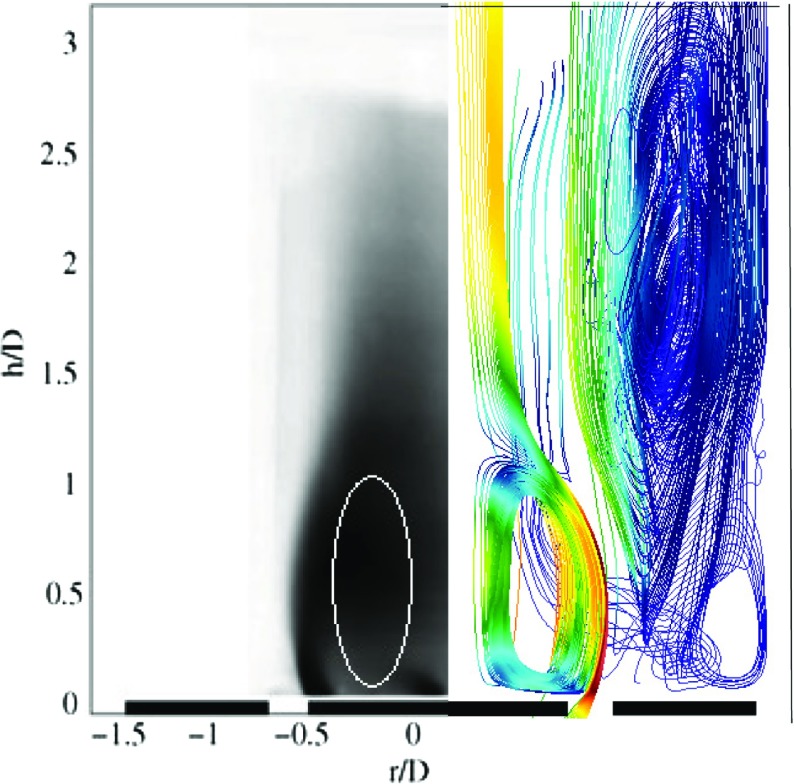
Streamlines from smoke visualization in the experiment (*left*) and flow streamlines of LES (*right*)

A preliminary evaluation of the fundamental flow features is also carried out. In the experiments, smoke visualization was used. An averaged smoke visualization image from the experiment along with the recirculation zone obtained from LES are shown in Fig. 7. The white line superimposed on the smoke visualization image shown in Fig. 7(left half) highlights the central recirculation zone of the bluff body. Good qualitative agreement between the experiment and the LES streamlines from the cold flow solution is obtained, with the central recirculation zone extending to around 35 mm downstream of the inlet plane.

**Fig. 8:**
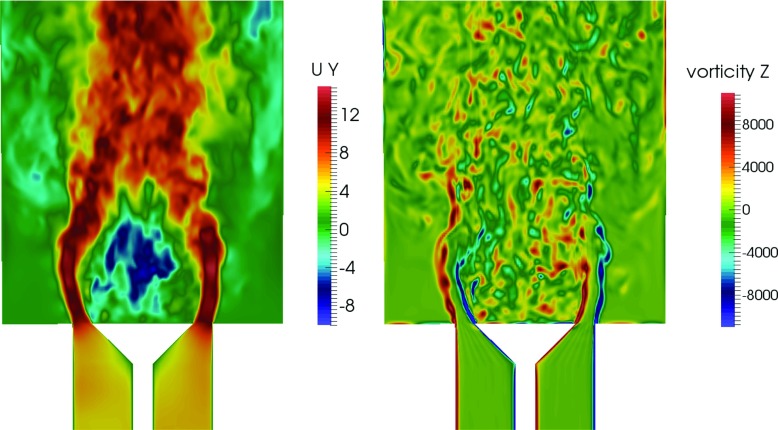
Instantaneous axial velocity (*left*) and radial component of vorticity (*right*)

**Fig. 9:**
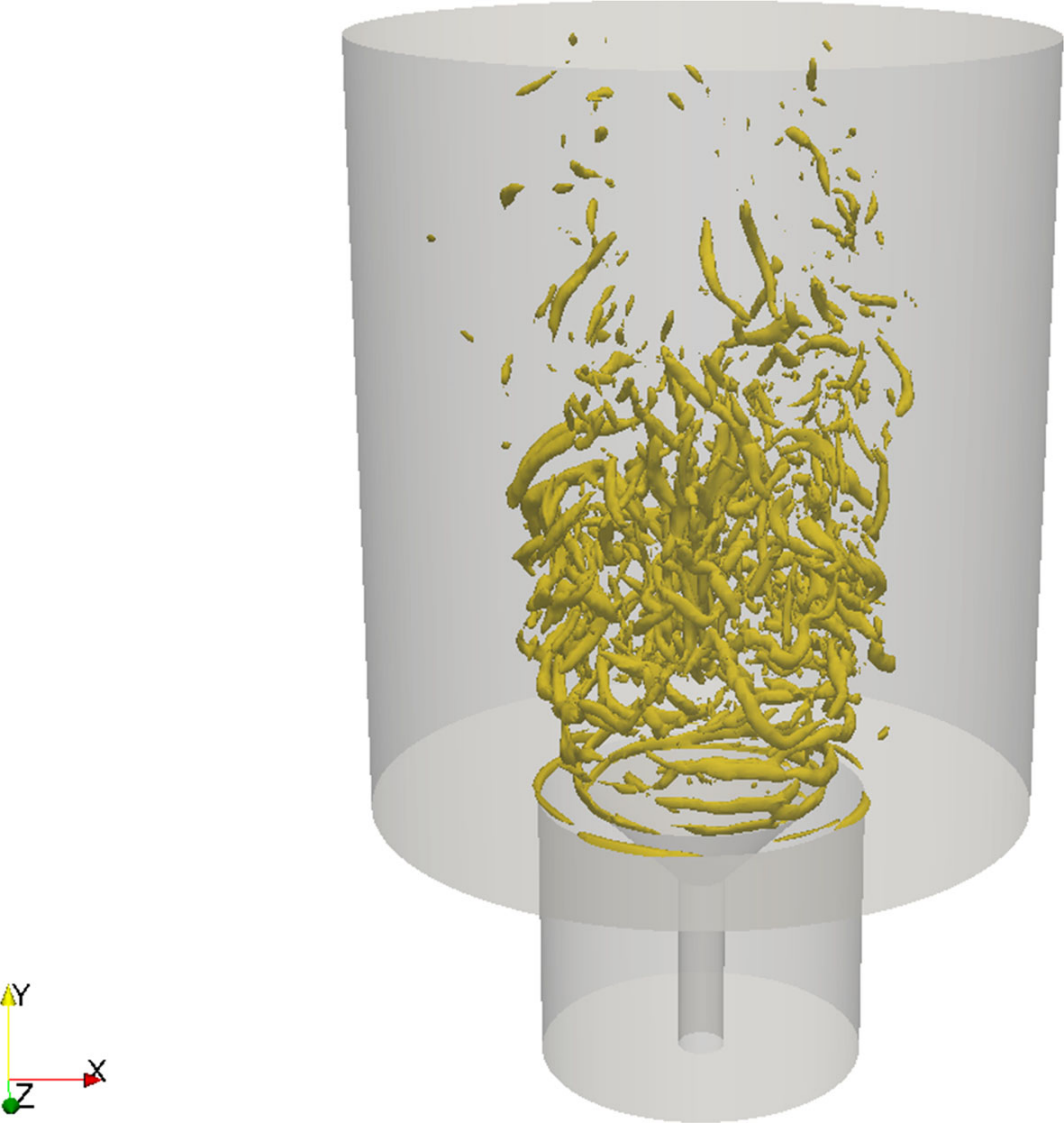
Isosurface of the Q-criterion at 1.2 × 10 ^7^ s ^−1^, showing the vortex structure at the shear layer and the wake

To illustrate more clearly the overall flow structure, snapshots of the instantaneous axial velocity and the radial component of the vorticity are shown in Fig. 8. The high velocity annular jets and the reverse flow in the central recirculation zone and the outer recirculation zone are evident. The vorticity contour suggests that Kevin-Helmholtz instabilities occur along the separated shear layer, as well as the presence of vortex merging and growth. At the end of the shear layer, some degree of vortex shedding can be observed. Breakdown of these vortices occurs further downstream of the shear layer and leads to smaller and less coherent structures. This can be observed in more detail by considering the Q-criterion on the isosurface Q = 1.2 × 10^7^ s ^−1^ (Fig. 9). The Q-criterion is defined based on the definition by Hunt et al. [37]. It can be seen that close to the bluff body, a helical (ring-like) like vortical structure is present due to the strong azimuthal variation in the velocity of the flow that emerges from the annulus. In the downstream region of the recirculation zone, vortex stretching can be observed as a result of intense mixing and vortex interaction.

### Assessment of subgrid-scale models and flow dynamics

In this section, the four subgrid models including the standard Smagorinsky model, Dynamic Smagorinsky model, WALE and the subgrid-scale TKE transport model are compared using an in-house implementation of each model in a local copy of OpenFOAM. This allows the models to be compared on the same basis. The choice of the subgrid scale model is important to capture the subgrid scale stresses in terms of the resolved velocity field in a manner such that the modelled stresses have to represent, as much as possible, the exact stresses. The performance of different models depends on the Reynolds number and filter size, and the preliminary validation performed in the previous section has established the feasibility of LES in predicting such a flow with reasonable accuracy. The comparison will be based on the results obtained using the dense mesh. Results corresponding to these models are compared.

**Fig. 10:**
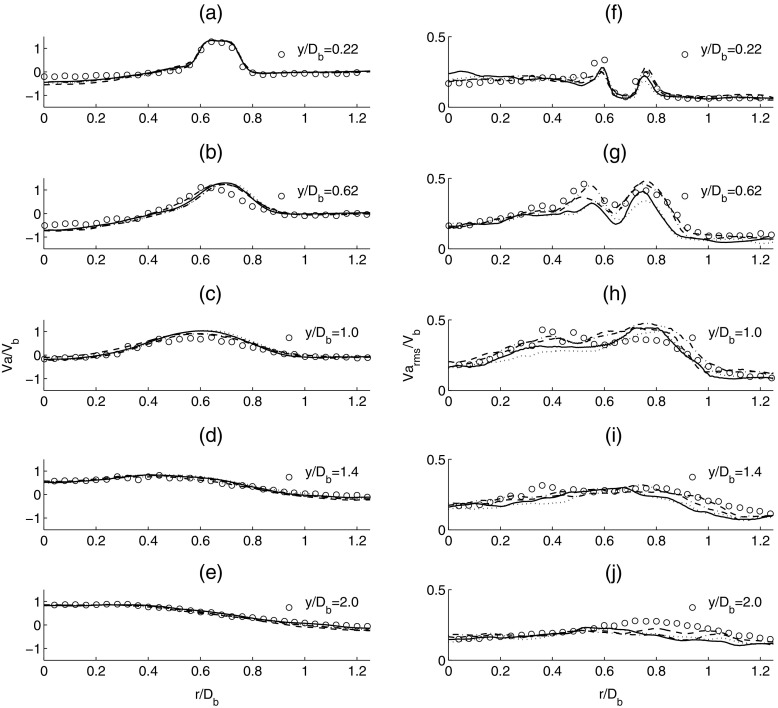
Time-averaged mean axial velocity (*left column*: **a-e**) and time-averaged rms axial velocity (*right column*: **f-j**). Experiment (*circle*), solid line (Smagorinsky), dashed line (Dynamic Smagorinsky), dotted (WALE) and dashed dotted line (subgrid TKE)

The comparison for these models is made against the mean and rms of both the axial and radial velocity profiles from the experiment. Figure 10 presents the comparison for the the mean (a-e) and rms (f-j) axial velocity profiles corresponding to the four different models at *y*/*D*_*b*_ of 0.22, 0.62, 1.0, 1.4 and 2.0 from the jet inlet. For the mean filtered axial velocity, results for all four models demonstrate good agreement with the experiment data. The mean axial velocity has a peak value of approximately 1.2 *V*_*b*_ after the exit of the bluff-body and a lowest value of approximately -0.5 *V*_*b*_ inside the recirculation zone at *y*/*D*_*b*_ = 0.62. After the exit of the bluff-body, the velocity profile becomes more uniform as vortex breakdown and mixing take place. For the rms axial velocity, it is evident that LES with different models reproduces the increase in axial velocity fluctuation in the shear layer at *y*/*D*_*b*_ = 0.22, 0.62 and 1.0 with reasonable accuracy, but gives a general trend of the inner shear layer being weaker than the outer shear layer. From the comparison, it appears that the Smagorinsky and WALE models predict a lower subgrid scale energy compared to the Dynamic Smagorinsky model and the subgrid-scale TKE model. This suggests that the both the Smagorinsky and WALE models are more dissipative than the Dynamic Smagorinsky model and the TKE model. Similar trends can be seen further downstream at *y*/*D*_*b*_ = 1.4 and 2.0.

**Fig. 11:**
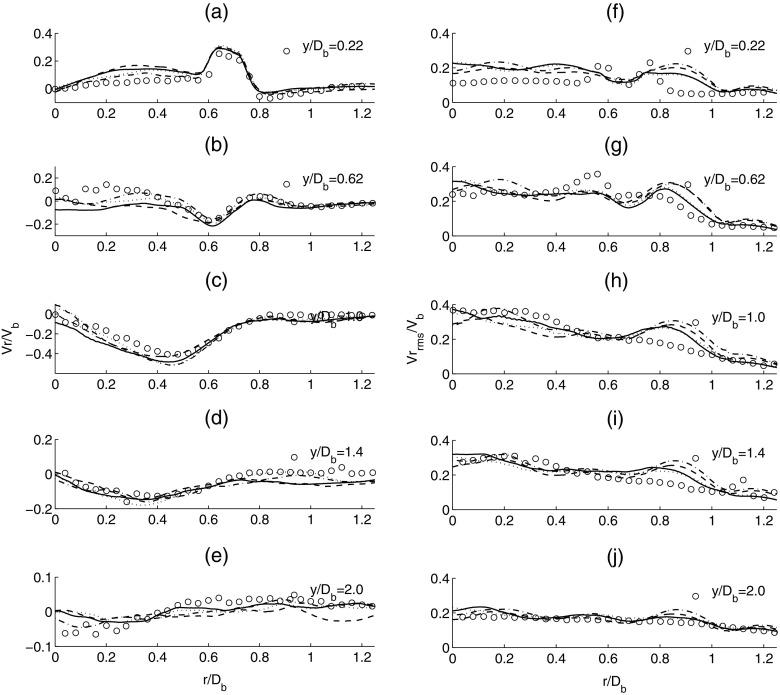
Time-averaged mean radial velocity (*left column*: **a-e**) and time-averaged rms radial velocity (*right column*: **f-j**). Experiment (*circle*), solid line (Smagorinsky), dashed line (Dynamic Smagorinsky), dotted (WALE) and dashed dotted line (subgrid TKE)

The corresponding profiles for the resolved mean and rms of the radial velocity are shown in Fig. 11. It can be seen that all subgrid scale models predict the mean filtered radial velocity very well. The increase in radial velocity between *r*/*D*_*b*_ = 0.6-0.8 at *y*/*D*_*b*_ = 0.22, and the dip in *r*/*D*_*b*_ = 0.6 and 0.5 at *y*/*D*_*b*_ 0.62 and 1.0, respectively can be seen clearly and display good agreement with the experiment. This demonstrates that the momentum transfer from the axial velocity component to the radial velocity component at the large scale in the asymmetric bluff-body flow is well captured. The general trend of the rms radial velocity at different axial locations is also predicted with reasonable accuracy using different models. The magnitudes of the rms radial velocity predicted by both the Dynamic Smagorinsky model and subgrid TKE model are once again higher than the predictions obtained using the Smagorinsky and WALE models.

**Fig. 12:**
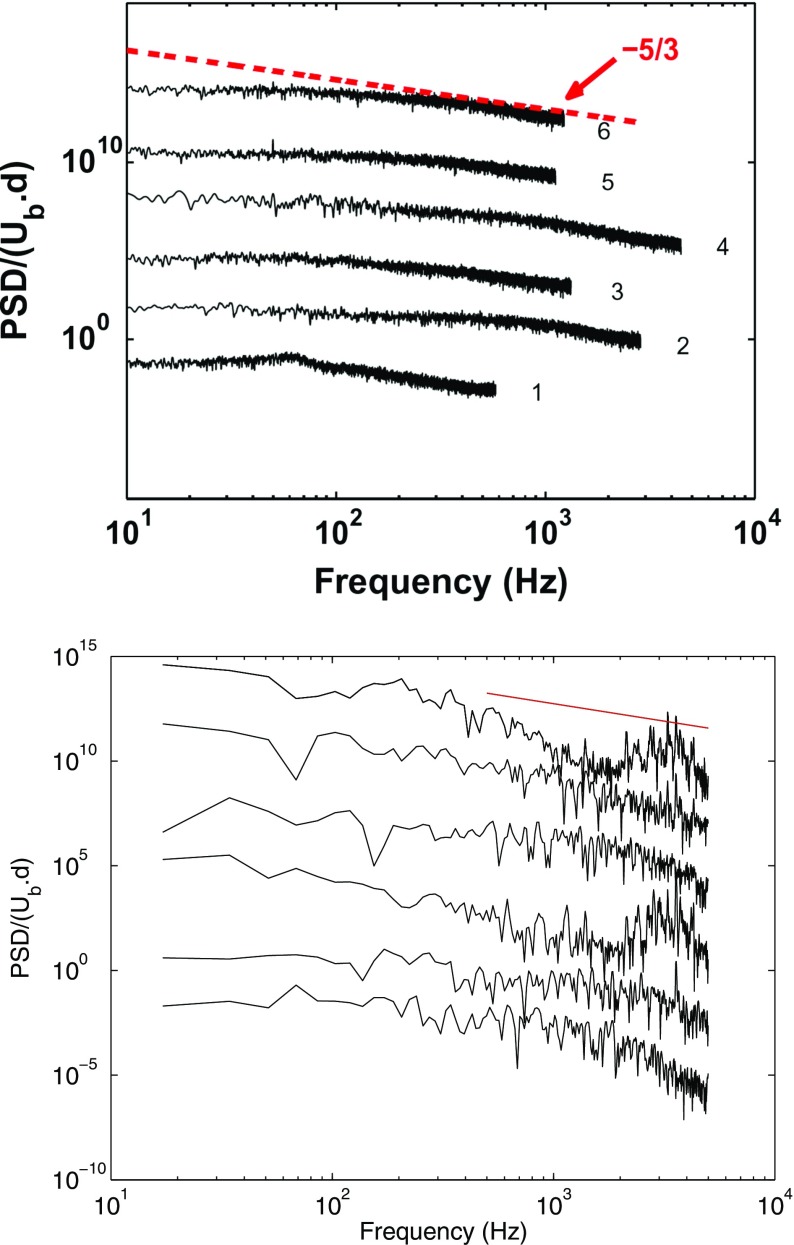
Spectra from experiment (*left*) and spectra from LES (*right*). Points 1-6 denotes the location of measurement shown in Fig. 13

**Fig. 13:**
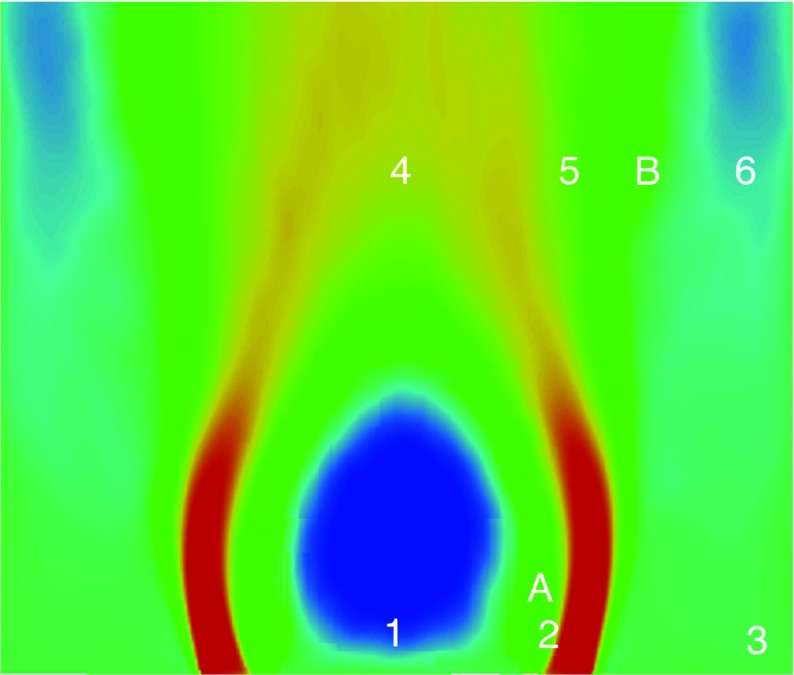
Locations of the measurements taken. Points 1-6 are at locations (*x*, *r*) = (5,0), (5,15), (5,30), (50,0), (50,15), (50,30) in mm, where *r* is distance from the axis and *x* axial distance from the bluff body

The turbulent spectra for the axial velocity obtained using the Dynamic Smagorinsky model is shown along with the spectra from the experiment in Fig. 12 for comparison. The axial velocity spectra for other subgrid-scale models are not presented as they exhibit a similar behaviour. The numbers on the right of the experimental spectra from 1-6 denote the locations where the measurements were taken [24]. These locations correspond to (*x*, *r*) = (5,0), (5,15), (5,30), (50,0), (50,15), (50,30) measured in mm, and are displayed in Fig. 13. It can be seen from the axial velocity spectra that the energy content increases at higher frequencies, particularly for locations 3 and 6, suggesting that small-scale structures are present in the outer recirculation region close to the wall. No distinct peak can be observed from the experiment or LES. Some rather distributed peaks at high frequencies can be seen in the LES spectra, which are due to the instability in the shear layer. This will be investigated in more detail in the following section. Also present is a small region with a slope of -5/3 from both experiment and LES, suggesting that the flow is fully turbulent in most of the region except perhaps inside the annular jet and at very short distances from the bluff body, where the turbulence levels and frequency content remain relatively weak.

**Fig. 14:**
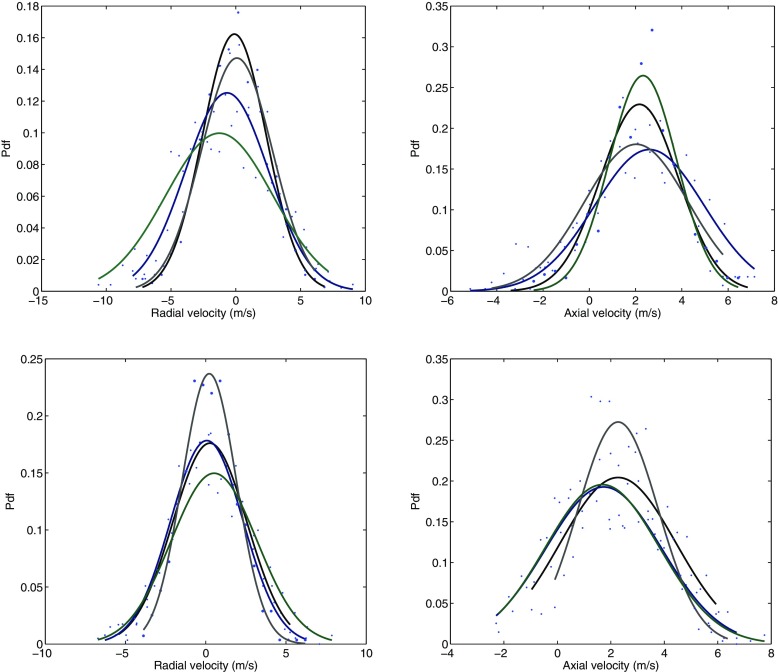
Probability density function of radial velocity (*left*) and axial velocity (*right*) taken at location A (*top*) and location B (*bottom*). Smagorinsky (*black*), Dynamic Smagorinsky (*blue*), WALE (*grey*) and Subgrid TKE (*green*)

The local flow behaviour in locations A (shear layer) and B (flow expansion region) is further examined. These locations are shown in Fig. 13, and correspond to (*x*, *r*) = (7,12.5), (50,20) in mm. The pdf of the radial and axial velocities for all four models taken at these locations are presented in Fig. 14, respectively. The pdf of the radial velocity component in location A exhibits a near-Gaussian distribution with a maximum spread (rms) of 10 m/s. The difference in rms values between these models lies within the range of 25 *%*. The spread over the mean value of nearly zero in the pdf suggests the presence of strong velocity fluctuations in the shear layer. The difference in the peak values of the pdf of different models ranges from a maximum of 16 *%* (Smagorinsky model) to minimum of 10 *%* (subgrid TKE model). A similar distribution can be seen for the axial velocity (right column) except that it is positively skewed towards a mean of approximately 2 m/s possibly due to the incoming flow from the annulus. At the downstream region (location B), the pdf for the radial velocity exhibits similar characteristics at location A, but with a lower rms and an increase in the probability of the mean value. These indicate that the velocity fluctuations in location B are smaller than in the shear layer. From the pdf of the axial velocity, a slight decrease in the mean velocity can be seen, and a wider spread is present. The pdf taken from different models displays a satisfactory agreement in terms of the rms and probability for both the axial and radial velocities at location B.

**Fig. 15:**
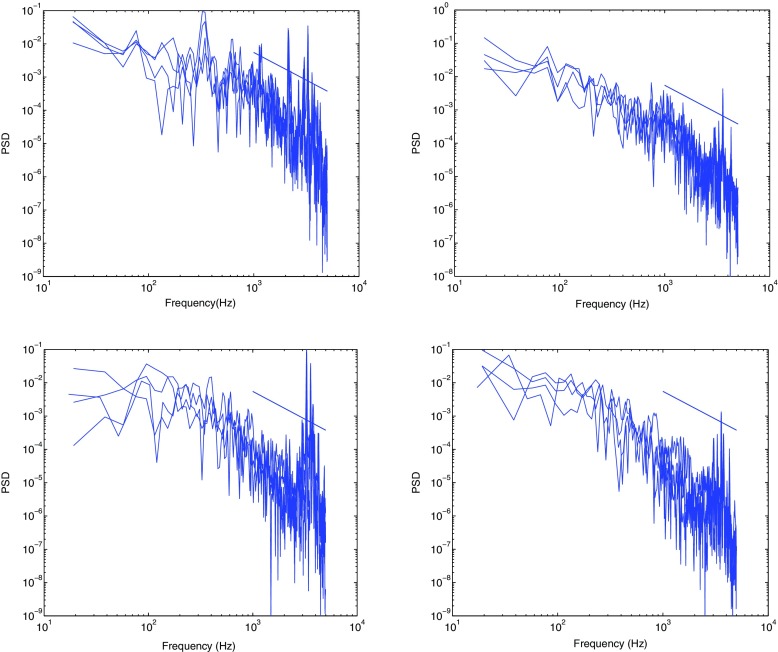
Spectra of radial velocity (*left*) and axial velocity (*right*) taken at location A (*top*) and location B (*bottom*)

Shown in Fig. 15 are the spectra of the fluctuating radial and axial velocities at locations A and B for different models. These spectra are plotted to identify the presence of flow instabilities close to the annular opening and the shear layer, as well as in the wake region of the enclosure. At location A (top row Fig. 15), distributed spectral peaks can be seen at frequencies 1100-1200 Hz and at 2000-3000 Hz, and weaker peaks corresponding to lower frequency range of 76-86 Hz and 300-360 Hz are also present. These peaks are consistent for all subgrid scale models. The peaks are however less pronounced in the spectra of the radial velocity in the downstream region (location B). The peak at approximately 1100 Hz and and its higher harmonics indicate Kevin-Helmholtz instability of the axisymmetrical flow at a Strouhal number of St _*KH*_ = *f*_*KH*_ *D*_*b*_/*U*_*b*_ =(1100)(0.03)/10 = 3.3. This Strouhal number is within the expected range for a shear-layer instability in a symmetrical bluff-body flow at Re ≈ 15000, as demonstrated by Sakamoto and Haniu [9]. The peak at approximately 350 Hz may indicate the presence of ring vortices at the edge of the bluff-body, which also been observed earlier by Broze and Hussien [38] in a pulsated jet. This frequency of this instability is known to be dependent on the lengthscale of the vortex structure, which is in turn governed by the annular opening of the enclosure. The weaker lower frequency flow instability at 75 Hz, however, is associated with the helical vortex shedding induced by the toroidal vortex core in such a flow configuration [12]. The Strouhal number corresponding to this process is St _*HS*_ = *f*_*HS*_ *D*_*b*_/*U*_*b*_ =(75)(0.03)/10 = 0.23. This Strouhal number is also observed for helical shedding in axisymmetric bluff bodies [9, 12], and lies within the appropriate range. Nevertheless, interaction of the Kelvin-Helmholtz instabilities of the shear layer with a shedding-type instability can cause large fluctuations of the reattachment length in the outer recirculation zone [24]. The Strouhal number corresponding to the reattachment length oscillation, as reported by Cole and Glauser [39], is also around 0.3, which lies very close to the frequency of helical shedding. Whether the low frequency is caused purely by helical shedding, or reattachment length fluctuation or a consequential effect of both, remains uncertain at this point. While central jet precessing is also expected to be present in such a flow configuration, at least for the lower range of Reynolds number, the characteristic frequencies associated with these processes are very low, at 5-10 Hz. To capture this frequency, a very large number of cycles is required and therefore it is challenging to investigate using LES.

As shown in the right column of Fig. 15, peaks associated with flow instability are less clear in the spectra of both the axial velocities taken at locations A and B, except for the frequency at 75 Hz. It also been argued that the spectra of the radial velocity fluctuations are more effective than the streamwise velocity in detecting low frequencies flow instabilities [39] which appears to be the case here. The absence of any dominant spectral peaks at location B demonstrates that the flow is well-mixed with homogeneous vortical structures present in the downstream region. The spectra reveals multiple peaks at high frequency. To identify the deterministic spatial structures that are responsible for a distinct fluid-mechanical instability in such a compounded flow, a detailed analysis using modal decomposition methods [40, 41] would be useful.

**Fig. 16:**
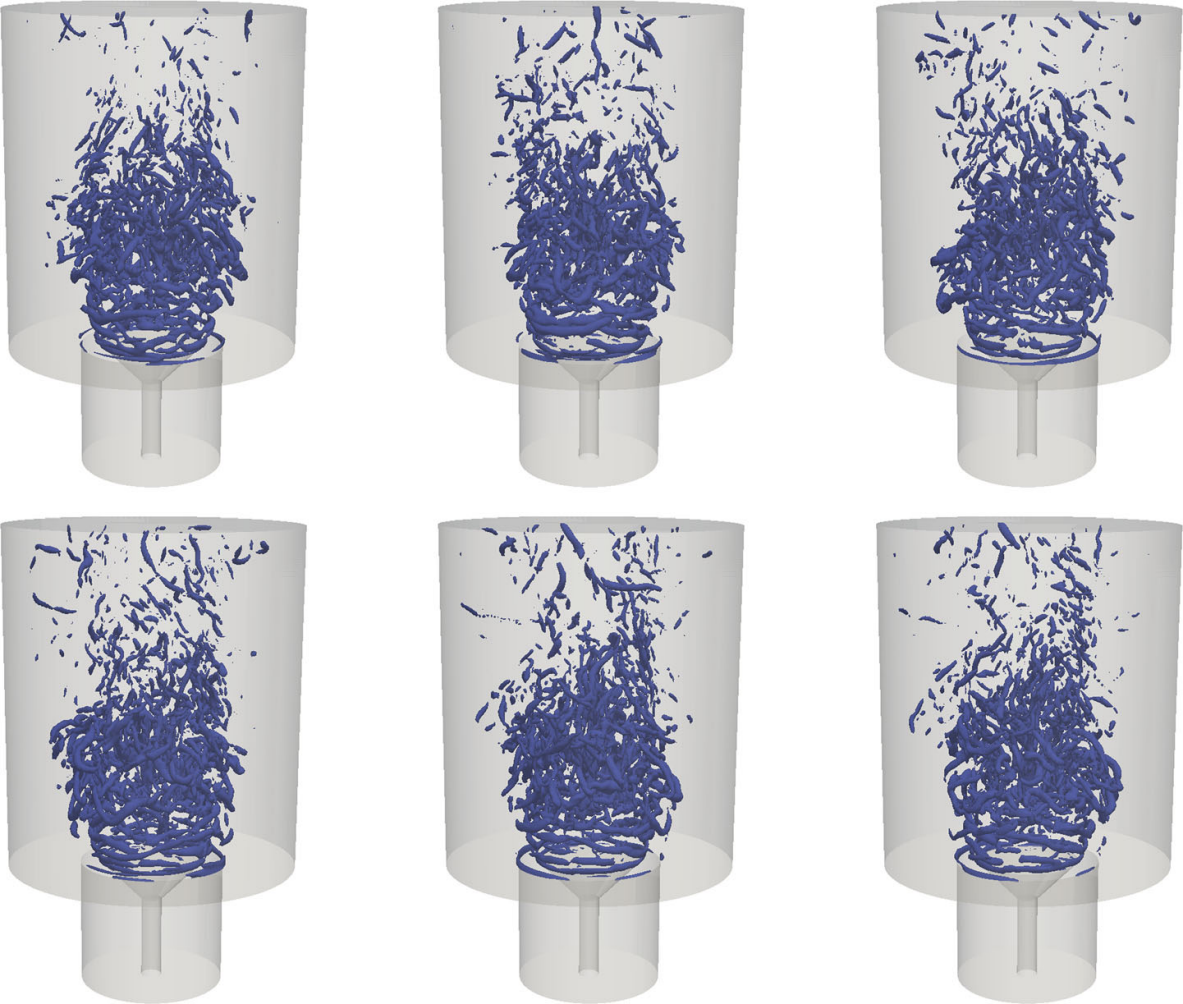
Six instantaneous snapshots of isosurfaces at Q = 1.2 × 10 ^7^ s ^−1^ showing the vortex structure of the flow

To highlight the more prominent features of the vortical structures, the Q-criterion [37] at isosurface Q = 1.2 × 10^7^ s ^−1^ is presented in Fig. 16. The separation time between the each snapshot is *t* = 0.0027 s, corresponding to a frequency of approximately 370 Hz. A total of 6 snapshots are shown, giving a duration of 0.0135 s (75 Hz). It is important to note that the turbulent flow is highly intermittent and does not contain a periodic nor a distinct oscillatory behaviour such as Karman vortex shedding. The choice of separation duration between each snapshot is hence made based on the characteristic frequencies observed in the spectra of the radial velocity at location A. It can also be seen that the most of the activity is concentrated in the flow closer towards the centreline: in the inner recirculation zone and the region of sudden expansion downstream of the stagnation zone.

Strong pulsation of the large-scale vortices manifests itself as ring-like structures close to the inlet of the annular jet due to shear layer instability. The vortex corresponding to this pulsation circulates around the central vortex core and has a tendency to be weighted towards a particular side of the ring structure. It can be observed that these helical structures start off being symmetrical close to the separation region at the bluff-body and align obliquely after a short distance downstream. This suggests the presence of a weak helical mode close to the inlet of the bluff-body and that the shear layer development is not perfectly symmetrical along the circumferential direction. The outer vortex ring is convected downstream, becomes highly distorted and eventually breaks up at an axial distance about approximately one bluff body diameter from the inlet. The breakdown of the vortex ring is characterized by the formations of hairpin vortex structures which are evident along the circumferential direction at the end of the annular jet. Such structures are known to be present in vortex shedding of axisymmetric bluff-body flows in the intermediate range of Reynolds number [12]. Some degree of asymmetric behaviour is again evident as a result of circumferential variation of the location where the horseshoe vortices are formed. Pairing of these horseshoe structures is also occasionally observed. Closer to the centreline, smaller vortices can be seen. These vortex tubes appear as the inner vortex ring contracts and disintegrates while it is being squeezed inside the central recirculation zone, and they tend to be randomly and intermittently orientated. The small-scale structures contribute to the broadband spectrum in the high frequency range, consistent with the those observed in the spectra.

From the previous observations, LES simulation is capable of adequately reproducing the mean and rms velocities, spectral peaks, vortex formation and the key features of the flow observed in the experiment. In this study, the choice of the model is found to have less effect on the large-scale (filtered) velocity as long as the most of the turbulent energy is well-resolved, but the rms velocity (subgrid-scale) is still dependent on the subgrid-scale models employed. The accuracy of the subgrid scale model would have important implications on processes occurring mainly at the molecular scale such as mixing and reactions, and with implications for predicting combustion oscillation. The consistency between the different models in terms spectral peaks and velocity pdfs has also been established, and therefore provides confidence in the use of LES in reproducing the appropriate flow dynamics.

## Conclusions

A numerical study was conducted using LES in order to analyze the flow behaviour and compare the results of different subgrid-scale models in an axisymmetric bluff-body flow. This flow serves as a model problem for combustors and the experiment data can help validate the LES results, which can be then used to gain a further understanding of the complex turbulent flow dynamics. Validation with experimental data and previous LES studies has demonstrated that the mesh resolution and numerical discretization are adequate for the current investigation. A higher mesh count provides a better result for the rms velocity fluctuations in LES but has less effect on the mean flow field. The recirculation length is also well predicted using LES. The performance of the four subgrid-scale models are then assessed using the Smagorinsky, Dynamic Smagorinsky, WALE and subgrid TKE models. In this work, most of the TKE (> 80 *%*) is resolved. Good comparisons of the mean axial and radial velocity profiles are obtained using LES, and the results are almost independent of the choice of the subgrid-scale models used. This demonstrates that at high Reynolds number, the main role of the subgrid-scale model is to provide a mechanism for dissipation. The general flow features including the shear layer, recirculation zone and sudden expansion are well captured. The rms velocity profiles however are found to be sensitive to the subgrid scale models employed although the discrepancy with the experiment is still quite small. It is found the rms velocity is generally overpredicted using the Dynamic Smagorinsky model and the subgrid TKE model. Conversely, the Smagorinsky and WALE models tend to underpredict the rms velocity profiles. It is also shown that the spectra from LES includes a region with a slope of -5/3 as seen in the experiment, and the velocity pdfs are consistent for the different models used. Broad spectral peaks at approximately 75-86 Hz, 350 Hz and 1200 Hz are found in the spectra of the radial velocity for all subgrid scale model used. These peaks are associated with the helical shedding and shear-layer instability, respectively. The vortex structure demonstrates that the flow instabilities are intermittent: ring-like vortices appear close to the bluff-body, shed obliquely, form horseshoe vortices and become less organized small-scale vortices downstream. This behaviour is consistent across all models.
